# Impact of hospital volume on clinical outcomes of hospitalized heart failure patients: analysis of a nationwide database including 447,818 patients with heart failure

**DOI:** 10.1186/s12872-021-01863-4

**Published:** 2021-01-25

**Authors:** Hidehiro Kaneko, Hidetaka Itoh, Haruki Yotsumoto, Hiroyuki Kiriyama, Tatsuya Kamon, Katsuhito Fujiu, Kojiro Morita, Nobuaki Michihata, Taisuke Jo, Norifumi Takeda, Hiroyuki Morita, Hideo Yasunaga, Issei Komuro

**Affiliations:** 1 The Department of Cardiovascular Medicine, The University of Tokyo Hospital, 7-3-1, Hongo, Bunkyo-ku, Tokyo, 113-8655 Japan; 2 The Department of Advanced Cardiology, The University of Tokyo, Tokyo, Japan; 3 The Department of Clinical Epidemiology and Health Economics, School of Public Health, The University of Tokyo, Tokyo, Japan; 4 The Department of Health Services Research, Faculty of Medicine, University of Tsukuba, Tsukuba, Japan; 5 The Department of Health Services Research, The University of Tokyo, Tokyo, Japan

**Keywords:** Hospital volume, Heart failure, Epidemiology

## Abstract

**Background:**

Hospital volume is known to be associated with outcomes of patients requiring complicated medical care. However, the relationship between hospital volume and prognosis of hospitalized patients with heart failure (HF) remains not fully understood. We aimed to clarify the impact of hospital volume on clinical outcomes of hospitalized HF patients using a nationwide inpatient database.

**Methods and results:**

We studied 447,818 hospitalized HF patients who were admitted from January 2010 and discharged until March 2018 included in the Japanese Diagnosis Procedure Combination database. According to the number of patients, patients were categorized into three groups; those treated in low-, medium-, and high-volume centers. The median age was 81 years and 238,192 patients (53%) were men. Patients who had New York Heart Association class IV symptom and requiring inotropic agent within two days were more common in high volume centers than in low volume centers. Respiratory support, hemodialysis, and intra-aortic balloon pumping were more frequently performed in high volume centers. As a result, length of hospital stay was shorter, and in-hospital mortality was lower in high volume centers. Lower in-hospital mortality was associated with higher hospital volume. Multivariable logistic regression analysis fitted with generalized estimating equation indicated that medium-volume group (Odds ratio 0.91, p = 0.035) and high-volume group (Odds ratio 0.86, p = 0.004) had lower in-hospital mortality compared to the low-volume group. Subgroup analysis showed that this association between hospital volume and in-hospital mortality among overall population was seen in all subgroups according to age, presence of chronic renal failure, and New York Heart Association class.

**Conclusion:**

Hospital volume was independently associated with ameliorated clinical outcomes of hospitalized patients with HF.

## Background

Heart failure (HF) is a major cause of unexpected hospitalization in developed countries, and hospitalization due to HF is also a great economic burden [1–5]. Therefore, providing appropriate acute healthcare services for hospitalized HF patients and building up an optimal medical care system is strongly required. From the perspective of medical care system, hospital volume attracts clinical interest. Previous studies showed that hospital volume was associated with outcomes of not only patients with advanced cardiovascular diseases [6–9] but also those requiring intensive hospital care [10–12]. Taking these into consideration, hospital volume may influence the outcomes of hospitalized patients with HF. Several preceding studies examined the relationship between hospital volume and outcomes in patients with HF [13–16]. For example, Kumbhani et al. analyzed the Get With The Guidelines-HF registry including elderly HF patients and showed that hospital volume was marginally associated with outcomes up to 6 months of follow-up [13]. However, most of the research in this field was conducted among patients in North America, and real-world data on the association of hospital volume with outcomes of hospitalized HF patients have been insufficient. In this study, we sought to explore the effect of hospital volume on in-hospital outcomes of patients with HF using a nationwide inpatient database in Japan.

## Methods

### Study design and data source

The Diagnosis Procedure Combination (DPC) database is a nationwide inpatient database in Japan that includes administrative claims and clinical data for approximately 8 million hospitalized patients per year from more than 1200 participating hospitals including all 82 academic hospitals. The hospitals participating in the DPC database were distributed across all 47 prefectures in Japan. The DPC database represents approximately 50% of all acute inpatients and covers more than 90% of all tertiary-care emergency hospitals in Japan. Academic hospitals are obliged to participate in this database. The participation in this database of community hospitals is voluntary [17–20]. The database collates main diagnoses, comorbidities present at admission, and complications during hospitalization using the International Classification of Disease and Related Health Problems 10th Revision (ICD-10) codes.

### Definition of hospital volume

We defined hospital volume as the total number of hospitalized patients with HF during the study period at each hospital. We categorized hospitals into tertile (low-, medium-, and high volume) groups, with approximately equal numbers of patients in each group. We divided study patients into three groups by this category.

### Statistical procedure

Continuous and categorical data were presented as median (interquartile range) and number (percentages), respectively. We compared continuous data using one-way analysis of variance. We performed chi-square analysis to compare categorical variables. Univariate logistic regression analysis was used to identify the association between each covariate (including hospital volume) and in-hospital mortality. The association of hospital volume with in-hospital mortality was evaluated using a multivariable logistic regression analysis with adjustment for other patient backgrounds, while also adjusting for within-hospital clustering using a generalized estimating equation [21]. We performed subgroup analysis according to age, presence of chronic renal failure, and New York Heart Association class. A probability value of < 0.05 was considered to indicate statistically significant difference. We performed statistical analysis using SPSS version 25 and STATA version 16.

## Results

### Study population

We studied 466,921 patients aged ≥ 20 years with New York Heart Association (NYHA) class ≥ II, admitted and discharged between January 2010 and March 2018 with the main discharge diagnosis of HF defined by ICD-10 codes I50.0, I50.1, and I50.9. Exclusion criteria were as follows: (1) length of hospital stay ≤ 2 days (n = 15,270) and (2) major procedures under general anesthesia (n = 3833). The final number of enrolled patients analyzed was 447,818.

### Baseline clinical characteristics

Table 1 presents the distribution of the numbers of the patients. We categorized centers into the following three groups according to the volume of hospitals where patients were admitted: low (number of patients < 598), medium (598–1009 patients), and high (> 1009). Median age was 81 years, and there was a statistical difference in the percentage of patients aged ≥ 85 years between three groups (p < 0.001). The proportion of patients having NYHA class IV symptom was 30.2% in low volume center, 32.5% in medium volume centers, and 35.5% in high volume center. There was a statistical significant difference in the proportion of patients having NYHA class IV symptom (p < 0.001). The proportion of intravenous use of inotropic agent, nitrate, and furosemide increased with hospital volume category.

**Table 1:** Characteristics of study population

	Hospital volume			
	Low (n = 149,182)	Medium (n = 149,507)	High (n = 149,129)	*p* value
Number of hospital	648	190	107	
Number of patients in each hospital	400 (276–504)	810 (692–919)	1405 (1158–1634)	
Age (years)	81 (72–87)	81 (72–87)	80 (71–86)	< 0.001
Age ≥ 85 years	53,068 (35.6)	51,603 (34.5)	48,549 (32.6)	< 0.001
Male sex	78,205 (52.4)	79,548 (53.2)	80,439 (53.9)	< 0.001
Body mass index (kg/m^2^)	22.1 (19.6–25.0)	22.2 (19.6–25.0)	22.0 (19.5–24.8)	< 0.001
Hypertension	96,539 (64.7)	102,989 (68.9)	101,547 (68.1)	< 0.001
Diabetes mellitus	45,518 (30.5)	48,361 (32.3)	47,140 (31.6)	< 0.001
Chronic renal failure	20,996 (14.1)	22,222 (14.9)	22,285 (14.9)	< 0.001
Chronic liver disease	6086 (4.1)	6185 (4.1)	5246 (3.5)	< 0.001
Chronic respiratory disease	17,282 (11.6)	17,707 (11.8)	15,752 (10.6)	< 0.001
Smoking	43,317 (29.0)	48,219 (32.3)	52,262 (35.0)	< 0.001
Myocardial infarction	3,699 (2.5)	4,182 (2.8)	4,636 (3.1)	< 0.001
Dilated cardiomyopathy	10,853 (7.3)	11,463 (7.7)	11,810 (7.9)	< 0.001
Ventricular tachycardia/ventricular fibrillation	5,751 (3.9)	6,664 (4.5)	7,874 (5.3)	< 0.001
Shock	2,918 (2.0)	3,167 (2.1)	2,680 (1.8)	< 0.001
Anemia	21,466 (14.4)	24,775 (16.6)	25,373 (17.0)	< 0.001
Barthel Index	65 (15–100)	65 (15–100)	55 (5–100)	< 0.001
New York Heart Association				< 0.001
Class II	46,002 (30.8)	43,519 (29.1)	40,294 (27.0)	
Class III	58,106 (38.9)	57,366 (38.4)	55,967 (37.5)	
Class IV	45,074 (30.2)	48,622 (32.5)	52,868 (35.5)	
*Medications within two days after admission*				
Beta blocker	44,322 (29.7)	48,517 (32.5)	55,547 (37.2)	< 0.001
Renin-angiotensin system inhibitor	50,814 (34.1)	53,377 (35.7)	62,167 (41.7)	< 0.001
Angiotensin converting enzyme inhibitor	21,565 (14.5)	23,160 (15.5)	26,987 (18.1)	< 0.001
Angiotensin II receptor blocker	30,206 (20.2)	31,180 (20.9)	36,480 (24.5)	< 0.001
Mineralocorticoid receptor antagonist	44,609 (29.9)	47,290 (31.6)	53,716 (36.0)	< 0.001
Intravenous inotropic agent	24,788 (16.6)	25,138 (16.8)	28,310 (19.0)	< 0.001
Intravenous nitrate	26,825 (18.0)	31,741 (21.2)	34,999 (23.5)	< 0.001
Intravenous furosemide	97,917 (65.6)	101,240 (67.7)	105,515 (70.8)	< 0.001

Data are expressed as median (interquartile range) or number (percentage)

### Procedures during hospitalization

Table 2 summarized procedures and outcomes during hospitalization. There were statistically significant differences in the proportion of patients requiring respiratory support, hemodialysis, and intra-aortic balloon pumping, medical cost, lengths of hospital stay, and in-hospital mortality (all, p < 0.001). The proportion of patients requiring respiratory support and intra-aortic balloon pumping increased with hospital volume category. Medical cost increased, whereas length of hospital stay was reduced with hospital volume. Finally, in-hospital mortality decreased with hospital volume.

**Table 2:** Procedures and clinical outcomes during hospitalization

	Hospital volume			
	Low (n = 149,182)	Medium (n = 149,507)	High (n = 149,129)	*p* value
Respiratory support	19,689 (13.2)	23,151 (15.5)	27,814 (18.7)	< 0.001
Hemodialysis	3594 (2.4)	3909 (2.6)	3882 (2.6)	< 0.001
Intra-aortic balloon pumping	916 (0.6)	1073 (0.7)	1186 (0.8)	< 0.001
Extra-corporeal membrane oxygenation	132 (0.1)	160 (0.1)	158 (0.1)	0.200
Cost (JPY)	712,555 (474,773–1,144,035)	743,775 (504,863–1,165,348)	755,800 (519,243–1,181,185)	< 0.001
Cost (USD)	6556 (4368–10,525)	6843 (4645–10,721)	6953 (4777–10,867)	< 0.001
Length of hospital stay (days)	18 (11–29)	17 (11–28)	16 (11–25)	< 0.001
In-hospital death	11,068 (7.4)	10,067 (6.7)	9687 (6.5)	< 0.001

Data are expressed as median (interquartile range) or number (percentage)

### Impact of hospital volume on in-hospital mortality

Table 3 showed the result of the logistic regression analyses. Univariate logistic regression analysis showed that medium-volume group (Odds ratio 0.90, p < 0.001) and high-volume group (Odds ratio 0.87, p < 0.001) had lower in-hospital mortality compared to the low-volume group. The multivariable logistic regression analysis fitted with a generalized estimating equation for in-hospital mortality also showed that medium-volume group (Odds ratio 0.91, p = 0.035) and high-volume group (Odds ratio 0.86, p = 0.004) had lower in-hospital mortality compared to the low-volume group.

**Table 3:** Determinants of in-hospital death

	Univariate logistic regression analysis			Multivariable logistic regression fitted with generalized estimating equation		
	Odds ratio	95% Confidence interval	*p* value	Odds ratio	95% Confidence interval	*p* value
*Hospital volume*						
Low	Reference			Reference		
Medium	0.90	0.88–0.93	< 0.001	0.91	0.83–0.99	0.035
High	0.87	0.84–0.89	< 0.001	0.86	0.78–0.95	0.004
Age (years)	1.05	1.05–1.06	< 0.001	1.04	1.04–1.04	< 0.001
*Sex*						
Female	Reference			Reference		
Male	0.86	0.84–0.88	< 0.001	1.27	1.23–1.32	< 0.001
Body mass index (kg/m^2^)	0.90	0.90–0.90	< 0.001	0.95	0.95–0.96	< 0.001
Hypertension	0.43	0.42–0.44	< 0.001	0.53	0.50–0.55	< 0.001
Diabetes mellitus	0.78	0.76–0.80	< 0.001	1.03	0.99–1.06	0.120
Chronic renal failure	1.71	1.66–1.75	< 0.001	1.57	1.51–1.63	< 0.001
Chronic liver disease	1.33	1.26–1.40	< 0.001	1.45	1.36–1.55	< 0.001
Chronic respiratory disease	1.01	0.98–1.05	0.568	0.98	0.94–1.03	0.473
Myocardial infarction	1.93	1.83–2.04	< 0.001	1.46	1.35–1.57	< 0.001
Dilated cardiomyopathy	0.87	0.83–0.91	< 0.001	1.19	1.13–1.26	< 0.001
Smoking	0.68	0.66–0.69	< 0.001	0.88	0.85–0.92	< 0.001
*New York Heart Association*						
Class II	Reference			Reference		
Class III	1.92	1.85–1.99	< 0.001	1.68	1.58–1.79	< 0.001
Class IV	4.49	4.33–4.66	< 0.001	3.36	3.11–3.63	< 0.001
Ventricular tachycardia/ventricular fibrillation	1.77	1.69–1.85	< 0.001	1.97	1.83–2.11	< 0.001
Shock	5.76	5.49–6.04	< 0.001	3.20	2.85–3.60	< 0.001
Anemia	1.55	1.50–1.59	< 0.001	1.35	1.30–1.41	< 0.001
Barthel Index per 10	0.84	0.83–0.84	< 0.001	0.89	0.89–0.90	< 0.001
*Administration within two days*						
Beta blocker	0.61	0.59–0.63	< 0.001	0.94	0.90–0.97	< 0.001
Renin-angiotensin system inhibitor	0.41	0.40–0.42	< 0.001	0.62	0.60–0.65	< 0.001
Mineralocorticoid receptor antagonist	0.63	0.61–0.65	< 0.001	0.83	0.80–0.86	< 0.001
Intravenous inotropic agent	2.88	2.81–2.95	< 0.001	2.13	2.04–2.23	< 0.001
Intravenous nitrate	0.64	0.62–0.66	< 0.001	0.57	0.54–0.60	< 0.001
Intravenous furosemide	1.32	1.29–1.36	< 0.001	0.97	0.94–1.01	0.172

### Subgroup analysis

**Fig. 1:**
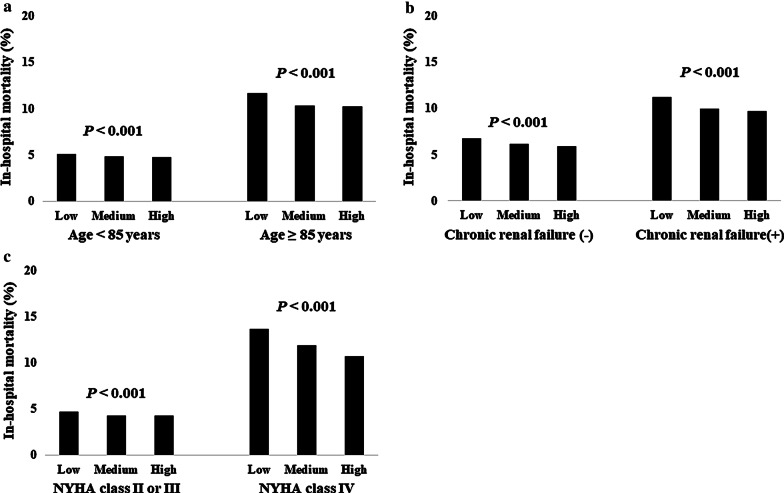
Subgroup analysis. Shows the result of the subgroup analysis according to age (**a**), presence of chronic renal failure (**b**), and New York Heart Association class (**c**)

Figure 1 presents the results of the subgroup analyses. Inverse association between hospital volume and in-hospital mortality was seen in all the subgroups including age (A), presence of chronic renal failure (B), and NYHA class (C).

## Discussion

Using a nationwide inpatient database of hospitalized patients with HF, we examined the influence of hospital-volume on clinical outcomes of HF patients. The key findings of the present study are as follows: (1) Patients in higher volume hospitals had more severe condition at admission. (2) Higher volume hospitals provided advanced cardiorespiratory support more aggressively. (3) Patients admitted in high volume hospitals had lower in-hospital mortality compared to those admitted to low volume hospitals even after adjustment for the covariates.

Baseline NYHA class increased with hospital volume, suggesting that patients admitted to higher volume centers were more severe than those admitted to low volume centers. Nevertheless, clinical outcomes were better in high volume centers than in low volume centers in association with early administration of medical agents and aggressive advanced cardiorespiratory supports.

In this study, we performed the multiple logistic regression analysis fitted with generalized estimating equation to identify the determinants of in-hospital death. Results of the multiple logistic regression analysis such as the association of chronic liver disease [22–24] and cigarette smoking (so called “smoker’s paradox” [25, 26]) with outcomes were concordant with preceding studies focusing on patients with HF, and our results demonstrated that hospital volume was independently associated with in-hospital mortality among patients hospitalized for HF.

Our findings were concordant with those in previous studies on the association between hospital volume and outcomes of HF patients, which also indicated that higher center volume was associated with lower mortality [14, 16]. On the contrary, the analysis of the Get With The Guidelines-HF registry which was linked to Medicare inpatient data of 342 hospitals showed that hospital volume was marginally associated with chronic phase outcomes, but not associated with in-hospital mortality [13]. Therefore, there has remained a room for further investigations in this field. Moreover, considering that previous studies examined patients limited to specific health insurance or community, our study has several advantages. Japan has a universal health care insurance system and patients are provided with equal health care access with equal fees which the Japanese government set. Further, our database is a nationwide inpatient database which could exclude region specific factors. Therefore, this study has strength in generalizability. Previous studies [14, 16] showed that higher hospital volume was associated with medical costs, and our study showed a similar trend. However, the difference in medical costs between low and high hospital volume hospitals was not so large in this study. Longer hospital stay in low volume hospitals and advanced procedures in high volume centers seemingly offset the difference in medical costs.

Several possible explanations can be suggested for the relationship between hospital volume and clinical outcomes. First, therapeutic management for hospitalized HF patients is complex and complicated. It requires a multidisciplinary team approach including primary care physicians, cardiologists, HF specialists, intensivists, cardiac surgeons, nurses, pharmacists, nutritionists, and physiotherapists who had not only professional training but also a great deal of experience in real-world clinical practice. Health care professionals in high volume centers may be more familiar with advanced treatment for hospitalized patients with HF. Therefore, rich experience in high volume centers may lead to better clinical outcomes. Second, various complications such as hemodynamic collapse, life-threatening arrhythmia, acute kidney injury, multiorgan dysfunction, stroke, and infection can occur during the clinical course of hospitalized patients with HF, and these complications require quick and appropriate management. A previous study reported that high volume centers could provide better management for complications in patients undergoing cardiac surgeries [27]. Therefore, better outcomes in high volume centers may attribute to better management for complications in patients with HF. Third, high volume centers may have more healthcare professional staffs and medical infrastructure. Abundant medical resources can also contribute to favorable outcomes in high volume centers. Finally, it could be also possible that better prognosis of HF patients leads to an increase in the number of patients through referral and results in further increase in hospital volume.

This study has clinical implications. As the prevalence of HF is increasing, nationwide actions for the optimal management of HF are required [28, 29]. From this point of view, establishing medical system for HF is important. The results of the present study suggest the importance of high volume centers for HF treatment. On the other hand, maintaining appropriate hospital access for patients with HF in each geographic region is essential because patients with acute HF requiring hospital admission need accessible hospitals which can provide emergency medical care. Therefore, low volume centers in each area is also indispensable. However, considering that the difference in in-hospital mortality according to hospital volume was seemingly pronounced in patients with severe conditions such as older age, chronic renal failure, and NYHA class IV, primary risk stratification for HF patients is important and we should prepare regional medical system for patients with HF. For example, it may be useful that emergency service or primary physicians determine a hospital where patients are transferred according to estimated severity of patients with HF.

There are several limitations in this study. We performed the multivariable logistic regression analysis to adjust for covariates, but a possibility of residual bias could not be eliminated. Because of the nature of the retrospective design, recorded diagnoses are commonly less well validated. Hospital volume was defined as the total number of hospitalized patients with HF during the study period at each hospital. Although some hospitals could have periods of time when they were not enrolling patients, we did not have enough information to assess this point in detail. Our database lacked data regarding blood pressure, heart rate (tachycardia and bradycardia), arrhythmia, HF etiology, left ventricular ejection fraction, diastolic function, right ventricular function, biomarkers such as brain natriuretic peptide, and other concomitant diseases, which can affect the clinical outcomes of hospitalized HF patients. In addition to these, other many factors are associated with the outcomes of HF patients. For example, serum uric acid level is also known to affect the prognosis of patients with HF [30, 31]. However, data on serum uric acid level were not available in this study. A previous study reported that higher hospital volume was associated with lower 6-month mortality and lower 6-month readmissions in patients with HF [13]. However, the long-term prognosis could not be assessed in this database.

## Conclusion

The analysis of a nationwide inpatient database including 447,818 hospitalized HF patients showed better clinical outcomes of HF patients in high volume centers compared to those in low volume centers. We believe that the results of this study are resourceful for preparing medical care system for hospitalized HF patients in the era of increasing prevalence of HF worldwide.

## Data Availability

The datasets analyzed during the current study are not publicly available due to contracts with the hospitals providing data to the database.

## Abbreviations

DPC: Diagnosis Procedure Combination
HF: Heart failure
ICD-10: International Classification of Disease and Related Health Problems 10th Revision
NYHA: New York Heart Association

## References

[1] Go AS, Mozaffarian D, Roger VL, Benjamin EJ, Berry JD, Borden WB, Bravata DM, Dai S, Ford ES, Fox CS, Franco S, Fullerton HJ, Gillespie C, Hailpern SM, Heit JA, Howard VJ, Huffman MD, Kissela BM, Kittner SJ, Lackland DT, Lichtman JH, Lisabeth LD, Magid D, Marcus GM, Marelli A, Matchar DB, McGuire DK, Mohler ER, Moy CS, Mussolino ME, Nichol G, Paynter NP, Schreiner PJ, Sorlie PD, Stein J, Turan TN, Virani SS, Wong ND, Woo D, Turner MB, American Heart Association Statistics C, Stroke Statistics S (2013). Heart disease and stroke statistics-2013 update: a report from the American Heart Association. Circulation.

[2] Chen J, Normand SL, Wang Y, Krumholz HM (2011). National and regional trends in heart failure hospitalization and mortality rates for Medicare beneficiaries, 1998–2008. JAMA.

[3] Jencks SF, Williams MV, Coleman EA (2009). Rehospitalizations among patients in the Medicare fee-for-service program. N Engl J Med.

[4] Storrow AB, Jenkins CA, Self WH, Alexander PT, Barrett TW, Han JH, McNaughton CD, Heavrin BS, Gheorghiade M, Collins SP (2014). The burden of acute heart failure on U.S. emergency departments. JACC Heart Fail..

[5] Fang J, Mensah GA, Croft JB, Keenan NL (2008). Heart failure-related hospitalization in the U.S., 1979 to 2004. J Am Coll Cardiol..

[6] Post PN, Kuijpers M, Ebels T, Zijlstra F (2010). The relation between volume and outcome of coronary interventions: a systematic review and meta-analysis. Eur Heart J.

[7] Russo MJ, Iribarne A, Easterwood R, Ibrahimiye AN, Davies R, Hong KN, Ascheim DD, Gelijns AC, Naka Y (2010). Post-heart transplant survival is inferior at low-volume centers across all risk strata. Circulation.

[8] Inohara T, Kohsaka S, Yamaji K, Amano T, Fujii K, Oda H, Uemura S, Kadota K, Miyata H, Nakamura M, Investigators JPR (2017). Impact of institutional and operator volume on short-term outcomes of percutaneous coronary intervention: a report from the Japanese Nationwide Registry. JACC Cardiovasc Interv.

[9] Vemulapalli S, Carroll JD, Mack MJ, Li Z, Dai D, Kosinski AS, Kumbhani DJ, Ruiz CE, Thourani VH, Hanzel G, Gleason TG, Herrmann HC, Brindis RG, Bavaria JE (2019). Procedural volume and outcomes for transcatheter aortic-valve replacement. N Engl J Med.

[10] Nathens AB, Jurkovich GJ, Maier RV, Grossman DC, MacKenzie EJ, Moore M, Rivara FP (2001). Relationship between trauma center volume and outcomes. JAMA.

[11] Light TD, Latenser BA, Kealey GP, Wibbenmeyer LA, Rosenthal GE, Sarrazin MV (2009). The effect of burn center and burn center volume on the mortality of burned adults–an analysis of the data in the National Burn Repository. J Burn Care Res.

[12] Worthington H, Pickett W, Morrison LJ, Scales DC, Zhan C, Lin S, Dorian P, Dainty KN, Ferguson ND, Brooks SC, Rescu I (2017). The impact of hospital experience with out-of-hospital cardiac arrest patients on post cardiac arrest care. Resuscitation.

[13] Kumbhani DJ, Fonarow GC, Heidenreich PA, Schulte PJ, Lu D, Hernandez A, Yancy C, Bhatt DL (2018). Association between hospital volume, processes of care, and outcomes in patients admitted with heart failure: insights from get with the guidelines-heart failure. Circulation.

[14] Madan S, Sims D, Saeed O, Patel SR, Shin JJ, Jorde UP (2017). Association of centre volume and in-hospital mortality in heart failure hospitalisations. Postgrad Med J.

[15] McAlister FA, Youngson E, van Diepen S, Ezekowitz JA, Kaul P (2018). Influence of hospital volume on outcomes for patients with heart failure: evidence from a Canadian national cohort study. Am Heart J.

[16] Joynt KE, Orav EJ, Jha AK (2011). The association between hospital volume and processes, outcomes, and costs of care for congestive heart failure. Ann Intern Med.

[17] Kaneko H, Itoh H, Yotsumoto H, Kiriyama H, Kamon T, Fujiu K, Morita K, Michihata N, Jo T, Morita H, Yasunaga H, Komuro I (2020). Characteristics and outcomes of super-elderly patients (aged>=90 years) hospitalized for heart failure—analysis of a nationwide inpatient database-. Circ Rep.

[18] Kaneko H, Itoh H, Yotsumoto H, Kiriyama H, Kamon T, Fujiu K, Morita K, Michihata N, Jo T, Takeda N, Morita H, Yasunaga H, Komuro I (2020). Association of cancer with outcomes in patients hospitalized for heart failure. Circ J.

[19] Kaneko H, Itoh H, Yotsumoto H, Kiriyama H, Kamon T, Fujiu K, Morita K, Michihata N, Jo T, Morita H, Yasunaga H, Komuro I (2020). Association between the number of hospital admissions and in-hospital outcomes in patients with heart failure. Hypertens Res.

[20] Itoh H, Kaneko H, Kiriyama H, Kamon T, Fujiu K, Morita K, Yotsumoto H, Michihata N, Jo T, Takeda N, Morita H, Yasunaga H, Komuro I (2020). Reverse J-shaped relationship between body mass index and in-hospital mortality of patients hospitalized for heart failure in Japan. Heart Vessels.

[21] Hanley JA, Negassa A, Edwardes MD, Forrester JE (2003). Statistical analysis of correlated data using generalized estimating equations: an orientation. Am J Epidemiol.

[22] Nikolaou M, Parissis J, Yilmaz MB, Seronde MF, Kivikko M, Laribi S, Paugam-Burtz C, Cai D, Pohjanjousi P, Laterre PF, Deye N, Poder P, Cohen-Solal A, Mebazaa A (2013). Liver function abnormalities, clinical profile, and outcome in acute decompensated heart failure. Eur Heart J.

[23] Inohara T, Kohsaka S, Shiraishi Y, Goda A, Sawano M, Yagawa M, Mahara K, Fukuda K, Yoshikawa T, West Tokyo Heart Failure Registry I (2014). Prognostic impact of renal and hepatic dysfunction based on the MELD-XI score in patients with acute heart failure. Int J Cardiol..

[24] Xanthopoulos A, Starling RC, Kitai T, Triposkiadis F (2019). Heart failure and liver disease: cardiohepatic interactions. JACC Heart Fail.

[25] Fonarow GC, Abraham WT, Albert NM, Stough WG, Gheorghiade M, Greenberg BH, O'Connor CM, Nunez E, Yancy CW, Young JB (2008). A smoker's paradox in patients hospitalized for heart failure: findings from OPTIMIZE-HF. Eur Heart J.

[26] Doi SA, Islam N, Sulaiman K, Alsheikh-Ali AA, Singh R, Al-Qahtani A, Asaad N, AlHabib KF, Al-Zakwani I, Al-Jarallah M, AlMahmeed W, Bulbanat B, Bazargani N, Amin H, Al-Motarreb A, AlFaleh H, Panduranga P, Shehab A, Al Suwaidi J, Salam AM (2019). Demystifying Smoker's paradox: a propensity score-weighted analysis in patients hospitalized with acute heart failure. J Am Heart Assoc.

[27] Ghaferi AA, Birkmeyer JD, Dimick JB (2009). Variation in hospital mortality associated with inpatient surgery. N Engl J Med.

[28] Komuro I, Kaneko H, Morita H, Isobe M, Nakayama H, Minematsu K, Yamaguchi T, Yazaki Y (2019). Nationwide actions against heart failure pandemic in Japan—what should we do from academia?. Circ J.

[29] Kaneko H, Morita H, Komuro I (2020). Beautiful harmony of the Japanese precious healthcare legacies for the new imperial era. Circ J.

[30] Anker SD, Doehner W, Rauchhaus M, Sharma R, Francis D, Knosalla C, Davos CH, Cicoira M, Shamim W, Kemp M, Segal R, Osterziel KJ, Leyva F, Hetzer R, Ponikowski P, Coats AJ (2003). Uric acid and survival in chronic heart failure: validation and application in metabolic, functional, and hemodynamic staging. Circulation.

[31] Pascual-Figal DA, Hurtado-Martinez JA, Redondo B, Antolinos MJ, Ruiperez JA, Valdes M (2007). Hyperuricaemia and long-term outcome after hospital discharge in acute heart failure patients. Eur J Heart Fail.

